# Case Report: Successful treatment of pediatric patients with acute generalized pustular psoriasis with secukinumab

**DOI:** 10.3389/fmed.2026.1867541

**Published:** 2026-09-10

**Authors:** Qiqi Liu, Lixin Chen, Bei Qin, Jia Lian, Qinfeng Li

**Affiliations:** Department of Dermatology, Children’s Hospital, Tianjin University/Tianjin Children’s Hospital, Tianjin, China

**Keywords:** children, generalized pustular psoriasis, histopathology, reflective confocal microscopy, secukinumab

## Abstract

Acute generalized pustular psoriasis (GPP) is a rare, distinct subtype of psoriasis characterized clinically by sterile pustules arising on an erythematous base. Pediatric GPP is associated with severe manifestations, high healthcare expenditures, and a great risk of mortality, severely affecting the quality of life of both children and their families. Secukinumab, a fully human monoclonal antibody that selectively targets IL-17A—a pivotal cytokine in psoriasis pathogenesis that promotes keratinocyte proliferation and inflammatory responses—has been approved for the treatment of moderate-to-severe plaque psoriasis in children aged 6–18 years. Here, we describe three pediatric patients with GPP who were successfully treated with secukinumab. To date, no secukinumab-related adverse effects have been observed among the three pediatric patients throughout their respective follow-up periods. Secukinumab may be an effective option for the treatment of GPP in children.

## Introduction

Acute generalized pustular psoriasis (GPP) is a rare, distinct subtype of psoriasis characterized clinically by sterile pustules arising on an erythematous base (1). The reported prevalence among Chinese pediatric psoriasis cohorts is below 1% (2). Unlike adult GPP, which frequently develops in individuals with a prior history of psoriasis vulgaris, pediatric GPP typically occurs without preexisting psoriasis vulgaris and is closely associated with mutations in genes such as IL36RN and AP1S3 (3). Pediatric GPP is associated with severe manifestations, high healthcare expenditures, and a great risk of mortality, severely affecting the quality of life of both children and their families (4). Common first-line systemic treatments for pediatric GPP include oral acitretin, cyclosporine, and methotrexate, which vary in efficacy and safety profiles but often require multiple interventions for short-term relief and long-term disease control (5). Secukinumab is a fully human IgG1 monoclonal antibody that selectively binds to the proinflammatory cytokine IL-17A and blocks the interaction between IL-17A and its receptor, thereby alleviating inflammatory responses in psoriasis (6). It has been approved in the United States, the European Union, and numerous other countries worldwide for the treatment of moderate-to-severe plaque psoriasis in pediatric patients aged 6–18 years (7). Robust and long-term therapeutic efficacy coupled with favorable safety profiles has been demonstrated in the clinical management of plaque psoriasis, psoriatic arthritis, ankylosing spondylitis and hidradenitis suppurativa (8, 9). Herein, we report three pediatric patients diagnosed with GPP in accordance with the diagnostic criteria established by the European Rare and Severe Psoriasis Expert Network (ERASPEN) and the Japanese Dermatological Association (JDA), all of whom achieved favorable clinical outcomes following treatment with secukinumab.

### Case 1

**Figure 1:**
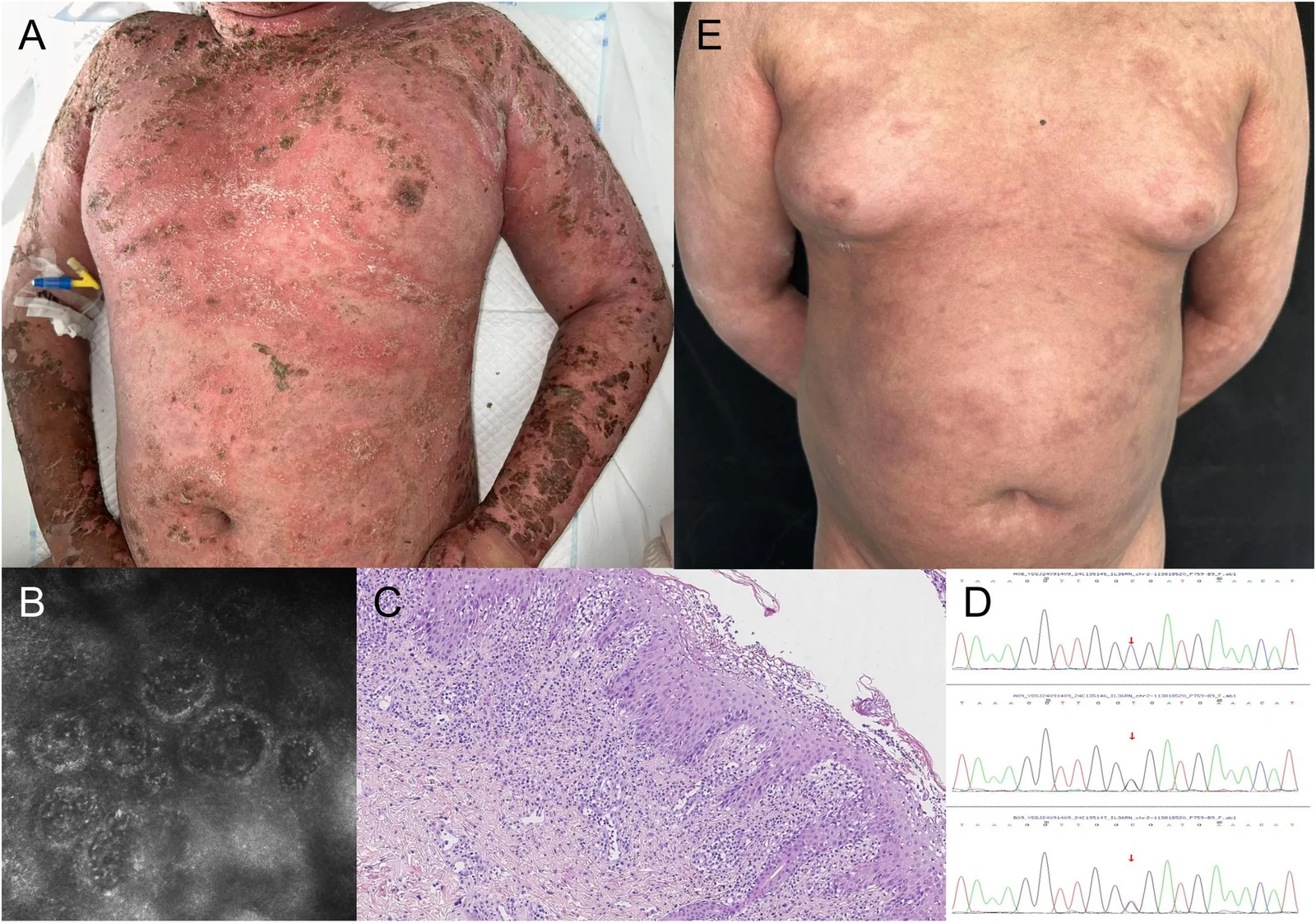
**(A)** Generalized pustular psoriasis affecting the trunk prior to initiation of secukinumab therapy. **(B)** RCM showed a large number of hyporeflective round or tubular structures within the basal cell ring, containing flowing hyperreflective granules. **(C)** Skin histopathology (anterior chest) demonstrated necrosis of the stratum corneum and superficial stratum spinosum with neutrophilic infiltration, extensive subcorneal spongiform pustule formation, acanthosis, uniform elongation of epidermal rete ridges, dilatation of dermal papillary blood vessels (HE, × 200). **(D)** Whole-exome sequencing identified a homozygous mutation (c.115+6T>C) in the IL36RN gene, resulting in a splice-site mutation; both parents carried heterozygous mutations at this locus (Case 1). **(E)** Week 1 secukinumab results.

An 11-year-old Chinese boy was admitted with generalized diffuse erythema for 10 days, followed by fever and pustules for 3 days. Dermatological assessment revealed diffuse erythema, edema, and numerous millet-sized pustules (some confluent into pustular lakes) on the scalp, face, trunk, extremities, dorsa of the hands and feet, and genitalia; some pustules had dried into yellow crusts (Figure 1A). Reflective confocal microscopy (RCM) of the erythematous lesion showed an increased number of epidermal scanning layers. Well-circumscribed hyporeflective cave-like areas were visible in the epidermis, which were densely populated with numerous medium-to-hyperreflective small round cells. Within the dermal papillary rings, many expanded low-refraction canalicular or structures, containing flowing hyperreflective granules. Irregularly distributed medium-to-hyperreflective small granular substances were noted around the dilated blood vessels (Figure 1B). Skin histopathology (anterior chest) demonstrated necrosis of the stratum corneum and superficial stratum spinosum with neutrophilic infiltration, extensive subcorneal spongiform pustule formation, acanthosis, uniform elongation of epidermal rete ridges, dilatation of dermal papillary blood vessels, sparse lymphocytic and neutrophilic infiltration in the superficial dermis, with an obscured dermo-epidermal junction (Figure 1C). Whole-exome sequencing identified a homozygous mutation (c.115+6T>C) in the IL36RN gene, resulting in a splice-site mutation; both parents carried heterozygous mutations at this locus (Figure 1D). The patient was diagnosed with generalized pustular psoriasis. The score of Generalized Pustular Psoriasis Area and Severity Index (GPPASI) was 33.2. The patient received local conventional therapies including medicinal wet compresses, medicated bath and topical glucocorticoids for 5 days. Despite standardized local therapy, the patient continued to develop new pustules, accompanied by progressive aggravation of generalized erythema and edema. No obvious local adverse reactions such as severe skin irritation or burning pain were observed. Since these local measures failed to halt disease progression, the patient received a 150 mg subcutaneous injection of secukinumab after written informed consent was obtained from the parents (Table 1). One day after the injection, no new pustules were observed, the existing pustules began to dry, and erythema severity diminished. Two days after the injection, the majority of the pustules were completely dry, the erythema evolved from bright red to dark red with a slight reduction in extent, and fine scaling appeared. One week after the injection, the pustular sites resolved to hyperpigmented macules, the erythema size decreased significantly, and the color faded to pale pink or a near-normal skin tone (Figure 1E). The GPPASI decreased by 90%. Subsequent dosing included weekly injections for doses 2–5, followed by injections every 4 weeks starting with dose 6. Complete clearance (100% reduction in the GPPASI) was achieved after 2 doses. A total of 8 doses were administered. During the 1-year follow-up period, disease recurrence was not observed.

**Table 1:** Clinical characteristics and treatment outcomes of pediatric GPP patients treated with secukinumab.

Case	Age/sex	Weight	Whole-exome sequencing	Systemic therapy	Secukinumab dose	Number of doses	GPPASI score (clearance rate)						Duration of active secukinumab treatment	Post-treatment follow-up period
							Baseline	48–72 h	1 Week	4 Week	24 Week	48 Week		
Case1	11/male	56 kg	IL36RN	None	150 mg weekly for 0–4 weeks, then 150 mg every 4 weeks	8	33.2	10.8 (67％)	3.1 (90％)	0 (100％)	0 (100％)	0 (100％)	16 weeks	1 year
Case2	11/male	65 kg	IL36RN	None		11	31.6	9.6 (70％)	5.1 (84％)	0 (100％)	0 (100％)	0 (100％)	28 weeks	2 years
Case3	10/male	31 kg	None	None	75 mg weekly for 0–4 weeks, then 75 mg every 4 weeks	9	32.2	10.8 (66％)	2.8 (91％)	0 (100％)	0 (100％)	Not reached48 Weeks	20 weeks	6 months

### Case 2

**Figure 2:**
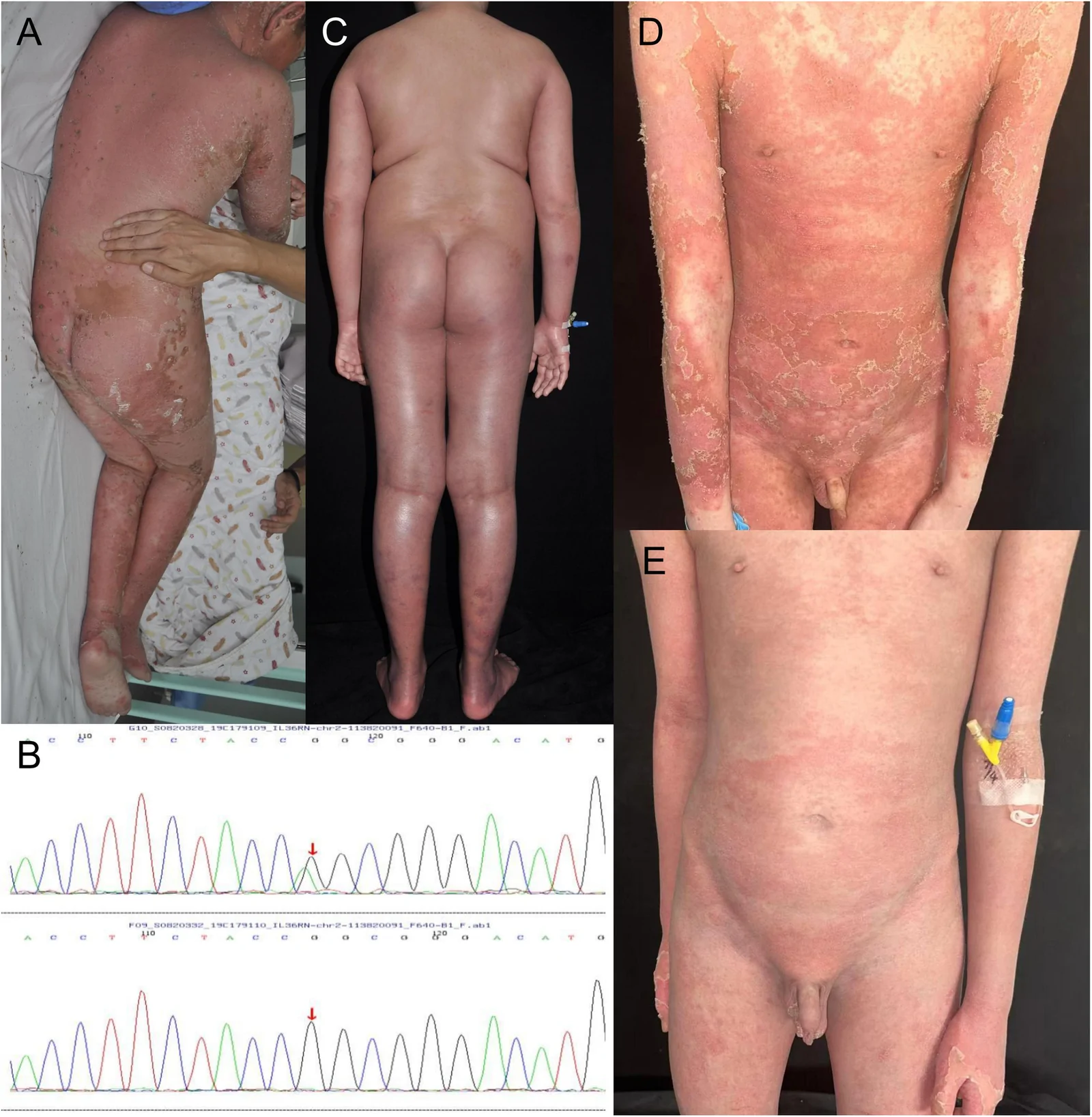
**(A)** Generalized pustular psoriasis affecting the trunk and limbs prior to initiation of secukinumab therapy. **(B)** Whole-exome sequencing identified a heterozygous mutation (c.115+6T>C) in the IL36RN gene; the father carried a heterozygous mutation at this locus (Case 2). **(C)** Week 1 secukinumab results (Case 2). **(D)** Generalized pustular psoriasis affecting the trunk and limbs prior to initiation of secukinumab therapy. **(E)** Week 1 secukinumab results (Case 3).

An 11-year-old Chinese boy was admitted with generalized erythema and pustules for more than 1 month, which worsened with fever for 1 week. Dermatological assessment revealed diffuse erythema, edema, and numerous millet-sized pustules on the scalp, face, trunk, extremities, dorsa of hands and feet, and genitalia; some pustules had dried into yellow crusts (Figure 2A). Whole-exome sequencing identified a heterozygous mutation (c.115+6T>C) in the IL36RN gene; the father carried a heterozygous mutation at this locus (Figure 2B). The patient was diagnosed with generalized pustular psoriasis. The score of GPPASI was 31.6. The patient received local conventional therapies including medicated bath, medicinal wet compresses, topical glucocorticoids, and laser irradiation for 7 days. There was transient mild fading of erythema; however, numerous pustules persisted and the lesion extent kept expanding. Transient local aggravation of erythema occurred following laser irradiation. Considering that local therapies could not control the systemic inflammatory status of generalized pustular psoriasis, all local therapeutic interventions were discontinued, and the patient received a 150 mg subcutaneous injection of secukinumab (Table 1). One week after the injection, pustular sites resolved to hyperpigmented macules, the lesion size decreased significantly, and the color normalized, with an 84% reduction in the GPPASI (Figure 2C). The subsequent dosing regimen was identical to that of Case 1. Complete disease resolution was achieved after 2 doses. A total of 11 doses were administered. During the 2-year follow-up period, disease recurrence was not observed.

### Case 3

A 10-year-old Chinese boy was admitted with generalized erythema, papules, and scaly plaques for 4 months and pustules for 20 days. Dermatological assessment revealed extensive diffuse erythema and papules involving 80% of the total body surface area on the head, face, trunk, and extremities, with pustules of varying sizes (some confluent into pustular lakes) and superficial white scaling (Figure 2D). No pathogenic genetic variants associated with the disease phenotype were identified by whole-exome sequencing. The patient had a prior history of plaque psoriasis. The diagnoses were as follows: 1. generalized pustular psoriasis; and 2. plaque psoriasis. The score of GPPASI was 32.2. The patient had intermittently used topical glucocorticoids during the previous course of plaque-type psoriasis. After progression to generalized pustular psoriasis, local conventional therapies including medicinal wet compresses, medicated bath, and laser irradiation were administered for 6 days. Only superficial scaling was mildly alleviated; nevertheless, abundant new pustules continued to emerge and skin lesions progressively worsened. The child reported burning pain at the laser-irradiated sites. Given insufficient therapeutic efficacy combined with laser-related local discomfort, all local conventional therapies were discontinued, and the patient received a 75 mg subcutaneous injection of secukinumab (Table 1). One week after the injection, the erythema size decreased, the color normalized, and the pustular sites resolved to hyperpigmented macules and minimal crusts (Figure 2E), with a 91% reduction in the GPPASI score. Subsequent treatment cycles were the same as in Case 1. Complete disease resolution was achieved after 3 doses. A total of 9 doses were administered. During the 6-month follow-up period, disease recurrence was not observed.

## Discussion

In Case 1, the short disease duration and absence of a positive family history created a diagnostic challenge in differentiating GPP from acute generalized exanthematous pustulosis (AGEP). AGEP is an uncommon but severe adverse cutaneous reaction that is most frequently drug-induced and is characterized by the abrupt development of diffuse edematous erythema followed by the formation of numerous sterile pustules and subsequent large-sheet desquamation within a short period (10). Histopathological examination of AGEP reveals keratinocyte necrosis with predominant dermal eosinophilic infiltration; in this pediatric case, however, inflammatory infiltrates were dominated by neutrophils. In addition, AGEP is self-limited and resolves with basic supportive care following drug discontinuation, whereas the patient failed to respond to standard supportive management. Bacterial culture of blister fluid yielded negative results, which ruled out infectious pustulosis from an etiological perspective. The child had no history of medication exposure, and cutaneous manifestations were fully consistent with generalized pustular psoriasis (GPP). Histopathological findings were concordant with the pathological features of GPP, and *in vivo* reflectance confocal microscopy imaging further validated the pathological diagnosis. Ultimately, identification of a pathogenic variant in the IL36RN gene provided definitive molecular evidence to establish the final diagnosis.

Cases 1 and 2 both presented with pathogenic variants in IL36RN and an acute onset, rapidly progressive disease with significant systemic involvement. Loss-of-function mutations in IL36RN lead to excessive activation of the IL-36 signaling pathway, clonal expansion of CD4^+^ T cells, and robust secretion of IL-17A (11). Accordingly, blockade of IL-17A constitutes a rational therapeutic strategy for GPP. Secukinumab has obtained regulatory approval for adult GPP in several countries (12). Although the efficacy of secukinumab in adults with GPP has been documented in several clinical studies, evidence in the pediatric population remains limited largely to case reports and small case series. Albela et al. reported a 4-year-old boy with a homozygous IL36RN mutation who achieved marked clinical improvement after treatment with secukinumab, which is consistent with our findings for Cases 1 and 2 (13). Moreover, a retrospective analysis involving 10 pediatric patients with GPP suggested durable clinical responses and favorable safety profiles, without the occurrence of serious adverse events (14). Mori Y et al. reported secukinumab as an effective and steroidsparing therapeutic option in pediatric GPP associated with IL36RN mutations (15). Although spesolimab, an IL-36 receptor-blocking monoclonal antibody, was approved in 2022 for the treatment of GPP in patients aged ≥12 years, clinical evidence for its use in younger children remains limited to case reports (16). The study conducted by Chen et al. demonstrated that five pediatric patients with GPP who failed to achieve long-term remission with prior therapies(including adalimumab, secukinumab, acitretin and topical drugs) exhibited favorable responses to a single dose of spesolimab (17). Peng et al. report the case of a low-weight, 5-year-old patient with GPP, who was refractory to multiple systemic agents, including adalimumab, but was successfully treated with spesolimab (18). Further multicenter studies are necessary to optimize pediatric dosing regimens and confirm long-term safety. For subjects with inadequate response to secukinumab, spesolimab serves as an alternative therapeutic option. Furthermore, the high cost of spesolimab imposes therapeutic constraints, which supports the selection of secukinumab as a safer and more accessible alternative from economic and practical perspectives. In view of the inadequate response to conventional therapy and the need for rapid disease stabilization, secukinumab was administered off-label after comprehensive counseling and obtaining informed consent. Both patients exhibited a rapid and substantial clinical improvement, with sustained remission and no adverse events during extended follow-up (2 years in Case 1 and 1 year in Case 2). These observations suggest that secukinumab may be a safe and effective therapeutic option for pediatric GPP driven by IL36RN mutations.

Case 3 developed GPP on the background of longstanding plaque psoriasis, which was likely precipitated by external triggers or insufficient prior disease control. Considering that secukinumab is already approved for the treatment of pediatric plaque psoriasis, its application in this patient was further justified.

Collectively, our findings indicate that secukinumab may represent a promising therapeutic option for pediatric GPP associated with IL36RN mutations as well as for GPP arising from poorly controlled plaque psoriasis. Rapid clinical resolution, typically within 48–72 h of the first dose, was observed across cases. This study was designed as a small-case series with inconsistent follow-up durations among enrolled patients. To date, the long-term safety profile and optimal dosing regimen of secukinumab for pediatric GPP remain inconclusive. Several investigators have reported atopic dermatitis-like eruptions arising from biologic therapy for psoriasis (19). More than 30 psoriasis patients treated with secukinumab have been documented to develop atopic dermatitis-like cutaneous adverse manifestations, presenting as eczematous lesions, allergic dermatitis-like exanthems or psoriasiform eruptions (20). This immune-rebalancing side-effect, known as immune drift, originates from Th1/Th2 immune imbalance and can trigger new-onset atopic dermatitis or exacerbate pre-existing atopic dermatitis (21). Close monitoring of cutaneous manifestations is therefore mandatory throughout secukinumab therapy. Furthermore, secondary loss of therapeutic response may occur with multiple biologic agents including secukinumab in adult plaque-type psoriasis. Data from the large adult psoriasis cohort of the British Association of Dermatologists Biologics and Immunomodulators Register (BADBIR) have demonstrated notable differences in 2-year adjusted effectiveness-related drug-survival across biologics; IL-17-pathway inhibitors exhibited a more pronounced decline in drug-survival with increasing prior biologic exposure (22). However, comparable real-world comparative drug-survival data for pediatric GPP are currently lacking. In the present small-case series, none of the three pediatric patients developed immune drift or secondary loss of response during follow-up. This finding should be interpreted with caution due to the small sample size and inconsistent follow-up durations. Long-term monitoring is therefore mandatory to evaluate sustained therapeutic efficacy among children with GPP treated with secukinumab.

## Data Availability

The original contributions presented in the study are included in the article, further inquiries can be directed to the corresponding author.
